# SELF-ACCEPTANCE: FORTITUDE FOR MIDDLE-AGED AND OLDER ADULTS, ENHANCING MENTAL WELL-BEING AMID RECESSION

**DOI:** 10.1093/geroni/igae098.3438

**Published:** 2024-12-31

**Authors:** Ilan Kwon

**Affiliations:** Our Lady of the Lake University, San Antonio, Texas, United States

## Abstract

Self-acceptance serves middle-aged and older adults to manage adversity and maintain mental well-being during Recession. Embracing both positive and negative aspects of oneself can cultivate a sense of self-worth, reduces vulnerability to external stressors, and bolster resilience. This positive attitude enables individuals to navigate adversity with greater adaptability and emotional stability and reinforce their coping mechanisms that enhance their capacity to manage adversity and maintain mental well-being despite economic hardship. Using the national data, Midlife in the United States (MIDUS 3, 2013-2014), this study aims to identify different patterns of socio-economic challenges experienced by middle-aged and older Americans (aged 50-64) during the 2008 recession and examine the interaction effect of the different types of challenging experiences and self-acceptance levels on later-life mental well-being. Latent class analysis of twenty-six socio-economic challenge items revealed five patterns of the Recession experiences: People encountering various difficulties, job transitions, financial hardships, homebuyers burdened with reduced debts, and those experiencing no significant challenges. Multiple regression results showed that people facing financial hardship and job mobility during the Recession reported heightened mental health issues compared to those with fewer challenges, even after controlling for other variables. Higher levels of self-acceptance correlated with better mental health outcomes. Notably, individuals with elevated self-acceptance demonstrated resilience in maintaining mental well-being despite economic adversity. By highlighting the protective role of self-acceptance, this study offers valuable insights into effective coping strategies and informs interventions aimed at bolstering the resilience and psychological well-being of middle-aged and older adults facing socio-economic challenges and adversity.

